# Mechanical performance of buried pipe under traffic load-internal pressure coupling action: Experimental and numerical study

**DOI:** 10.1371/journal.pone.0335464

**Published:** 2025-10-24

**Authors:** Changxi Shan, Wenhao Li

**Affiliations:** School of Civil Engineering, Chongqing University, Shapingba District, Chongqing, China; Dawood University of Engineering and Technology, PAKISTAN

## Abstract

With the accelerated progress of urbanization, there is an increasing occurrence of buried flexible water distribution pipelines subjected to high internal pressure and heavy loads. The probability of damage to these pipelines is magnified due to traffic load-internal pressure coupling action. Hence, investigating the mechanical performance of buried pipelines under such conditions is a topic of significant research importance. The pipe made of acrylate polymer blended with polyvinyl chloride resin for water supply (ABR pipe) is modified from PVC pipe, exhibiting high toughness, low temperature, and impact resistance. Experimental and numerical studies were conducted to investigate the mechanical performance of buried ABR pipe by applying traffic load, internal pressure, and a combined traffic load-internal pressure on the buried ABR pipe. The experimental and numerical anlyses were aimed to study the mechanical performance and deformation characteristics of the buried ABR pipe under varying loading conditions. The research results indicated that the mechanical performance of buried ABR pipe was superior, with the most critical sections occurring at 90° and 180°. The circumferential stress of the ABR pipe increased with the growth of internal pressure and traffic load, and the influence of internal pressure was significantly higher than that of traffic load. Additionally, with the increased pipe diameter to thickness ratio, the circumferential stress of the ABR pipe was significantly decreased. Furthermore, a theoretical calculation model for the buried ABR pipe under soil pressure, traffic load, and internal pressure was established based on the prism load method and Moore’s method. Finally, the circumferential stress calculation theory for buried ABR pipe was proposed based on safety factors. This theory was crucial for enhancing the safety and reliability of buried flexible water distribution pipelines under various load conditions.

## Introduction

Urban water distribution pipelines, typically buried along roadways, increasingly face challenges from high-frequency and heavy traffic loads [1]. These conditions pose significant risks to the structural integrity of the pipelines, especially when internal pressure is involved [2]. The radial deformation of buried pipelines is exacerbated by internal pressure, the structural integrity of pipelines is compromised, and the probability of pipeline failure is increased [3]. Furthermore, the secondary damage caused by the shell of pipe rupture and disconnection of pipe joints due to traffic load should be of concern. The cushion layer and backfilled soil around the pipeline are eroded due to the pipe leakage caused by secondary damage, resulting in reduced support and increased susceptibility to further damage [4]. Hence, understanding the mechanical performance of buried pipelines under traffic load becomes crucial for the safety assessment of urban water distribution systems.

Researchers have primarily studied the mechanical performance of buried pipelines under traffic loads in recent decades through theoretical analysis, prototype tests, and numerical simulation. The static analysis of buried pipelines under traffic load is addressed using the Boussinesq method [5] or spread angle method [6], pipeline longitudinal stress calculation model of Winkler foundation beam model, and pipeline circumferential stress calculation model based on Spangler-Iowa formula [7]. Dynamic analysis was mainly based on the Galerkin discretization method [8] and the Newmark-β method [9] The Fourier method has also been used to evaluate the impact of the wheel’s geometric shape and material properties on buried pipeline behavior [10].

Buried prototype tests represent the most intuitive approach to studying the effects of traffic loads. Researchers have conducted on-site experiments to investigate the mechanical performance of buried pipelines under dynamic and static traffic loads, considering the influence of pipeline burial depth [11] and diameter [12]. Considering the singularity and difficulty of the field prototype test, finite element analysis is commonly used to consider the complex response of the buried pipeline with multiple factors combined. Yang et al. [13] considered the effects of overloading, height differences, and bottom voids on the dynamic response of buried pipelines. Balkaya et al. [14] investigated the influence of uneven settlement of the cushion on the stress and strain of bell-and-spigot water supply pipelines. Zhang et al. [12] evaluated the axial strain of X65 pipelines by considering the nonlinear interaction between the pipeline and soil and accurately modeling the dynamic vehicle loading. However, most current studies have focused on concrete and PE pipelines and lack prototype buried tests for PVC pipelines.

With the development of Chinese PVC pipe industry technology, the pipe made of acrylate polymer blended with polyvinyl chloride resin for water supply(ABR pipe) was developed based on the PVC pipe, which solved the problems of low strength and poor toughness for PVC pipes. The ABR pipe was based on the blending of acrylate resin, polyvinyl chloride resin, thermal stabilizers, and other auxiliary materials, resulting in the acrylic super-strong molecular chain and the polymerization of acrylate-blended polyvinyl chloride material was obtained. These properties conferred high toughness, low-temperature resistance, and impact resistance to ABR pipes [15]. Shan et al. [16] conducted low-speed impact performance tests on ABR pipes to investigate the impact resistance of ABR pipes. Yang et al. [17] conducted pipe burst tests on ABR pipes with different pipe diameters to investigate the ultimate internal pressure bearing capacity. The ultimate internal pressure bearing capacity was increased by 14.1% compared with the PVC pipe. The test and analysis results indicated that the energy dissipation capacity, impact resistance, and mechanical performance of ABR pipes were significantly enhanced by adding materials such as acrylate polymer. The above studies have proved that ABR pipes have a broad application prospect for water supply and distribution projects. Nevertheless, the reliability and safety of buried pipeline networks have been put into higher demand with the increased urbanization level. Thus, the reliability and safety of buried ABR pipes under traffic load-internal pressure coupling action was significant for further study.

In this study, buried loading experiments were conducted on ABR pipe. The stress and strain of ABR pipe under traffic loads, internal pressure, and the traffic load-internal pressure coupling action were comparatively analyzed. The influence of the traffic load loading location on the mechanical response and deformation characteristics was investigated. The location of the critical cross-section under different loading conditions was determined. Three-dimensional numerical models were established using Abaqus finite element software to analyze the deformation characteristics of buried ABR pipes under parameters such as the location and magnitude of traffic loads, internal pressure, and the pipe diameter-thickness ratio. Furthermore, the safety factor theoretical calculation model for circumferential stress of buried ABR pipe was proposed based on the prism load method and Moore’s methods.

## Materials and experimental methods

### Experimental methods

**Fig 1:**
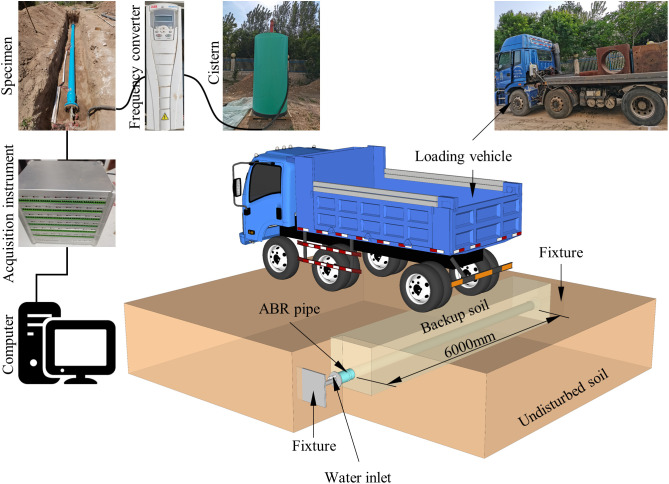
ABR pipe buried experimental design.

This study investigated the influence of traffic load and internal pressure on buried ABR pipes. Traffic load was applied to the buried ABR pipe through a static vehicle load to represent the traffic conditions accurately. Flanges were installed at both ends of the ABR pipe to seal it. After sealing, the internal pressure was applied to the ABR pipe by a water pump. The internal pressure magnitude was controlled by a frequency converter, and the real-time internal pressure was monitored through a data acquisition instrument. The trench for the buried ABR pipe should ensure that it can accommodate the pipe installation and provide sufficient support and space. According to Chinese code CJJ 101–2016 [18], the excavation depth and width of the trench were both 1.0 m. Considering the typical size of urban water supply pipes, the experiment selected ABR pipes with a length of 6 m and a nominal outer diameter of 200 mm. The chosen wheelbase of the static vehicle load was 1.2 m, and the load was concentrated on the rear wheels. There were four tires on the rear axle. The length and width of each tire were 0.6 m and 0.2 m, respectively. The experimental design described above is illustrated in Fig 1.

**Fig 2:**
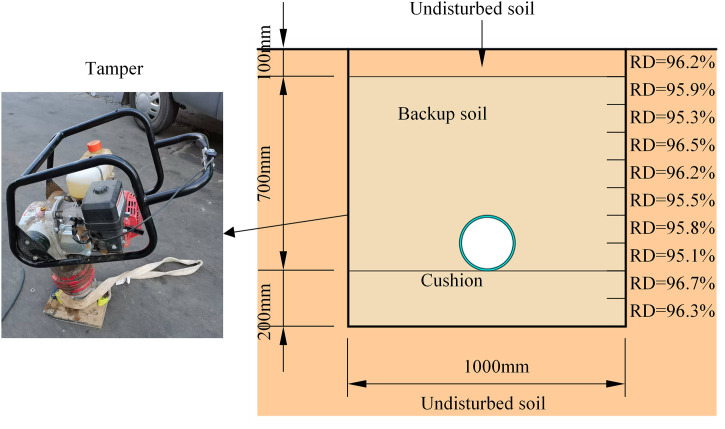
ABR pipe backfill. **(a)** Stress-strain curve. **(b)** Ring stiffness curve.

The trench backfilling is illustrated in Fig 2. The hard cushion layer was initially laid at the bottom for the trench stable pipe support. The ABR pipe was then laid on the cushion along the trench centerline. The rib angle portion within the supporting angle range beneath the ABR pipe was compactly backfilled to form a soil arch foundation to prevent pipe movement. Backfill materials included medium sand and backfill soil. The thickness of each backfill was 100 mm, and it was compacted with a tamper (Model W-100, 10 kN impact force, 600–700 impact frequency). The sand compaction degree was measured using the sand cone method during the backfilling process. When the medium sand was backfilled to 500 mm at the top of the ABR pipe, the 100 mm height of backfill soil was backfilled on the medium sand to prevent the wheel from damaging the backfilled sand and influencing the experimental results. Each 200 mm soil layer was compacted in 3 passes at 0.6 km/h speed, achieving ≥95% relative density(RD).

### Material properties

**Fig 3:**
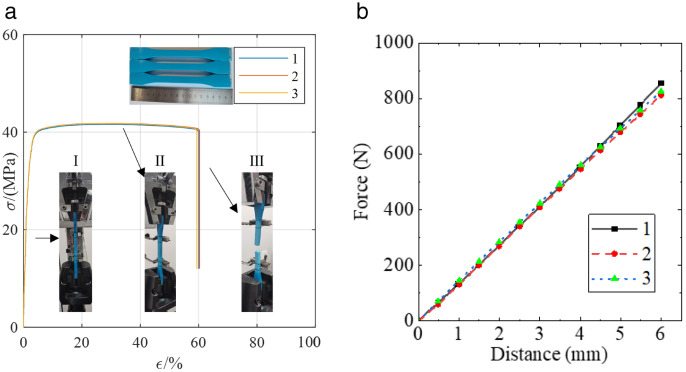
Stress-strain and ring stiffness curves of ABR pipes.

**Fig 4:**
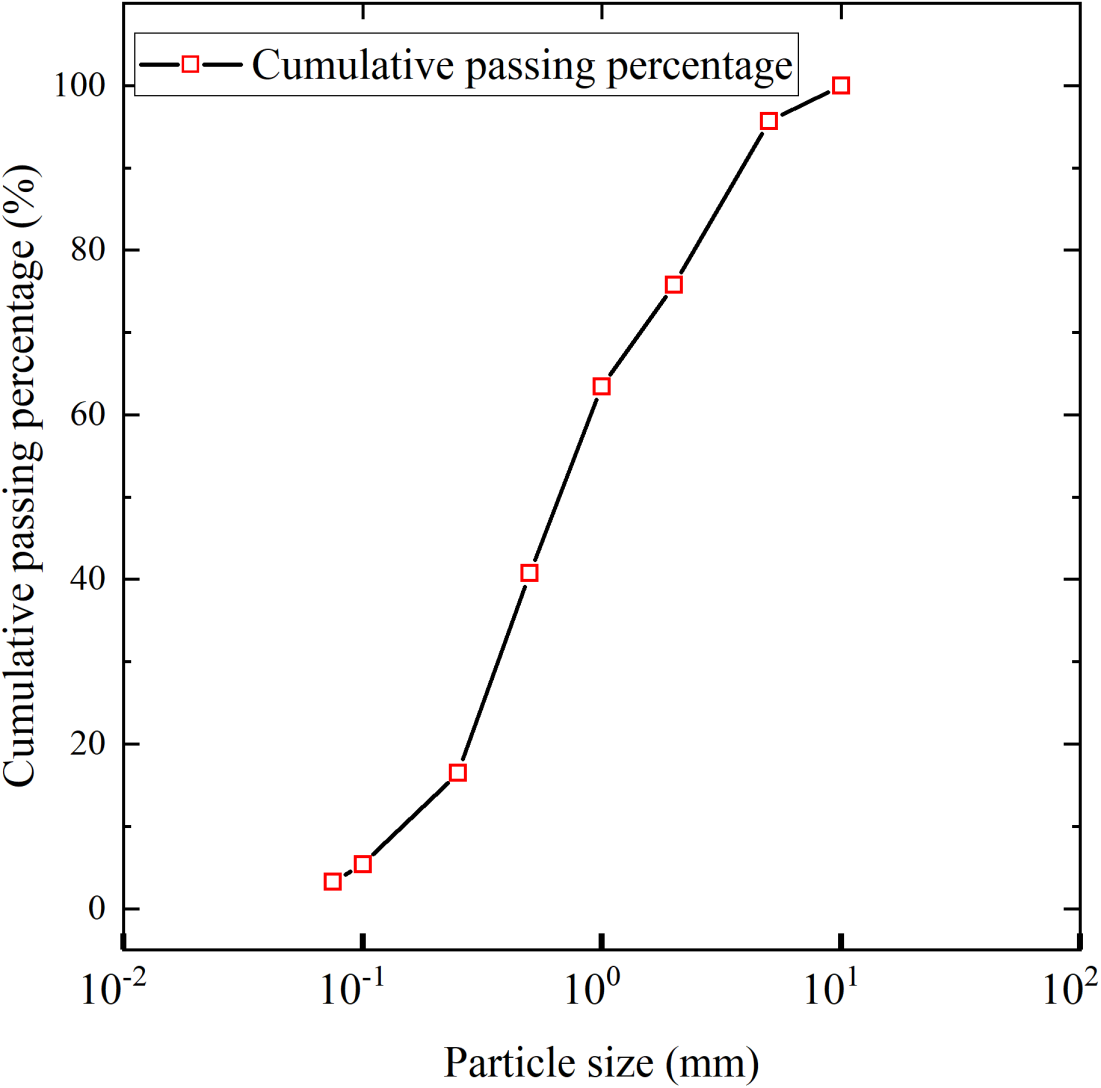
Grading curve of medium sand.

The ABR pipes used in the experimental were manufactured by Shandong Dongxin Plastic Technology Co., Ltd. The specifications of the ABR pipes were DN 200 × t 6.2 × PN 1.0. The yield strength, elasticity modulus, and elongation at the break of the ABR pipes were tested according to ASTM D638-14 standard [19]. The stress-strain curves of the ABR pipe are shown in Fig 3(a). The ring stiffness of the ABR pipes was tested according to the Chinese group standard T/SDAQI973–2022 [20], and the ring stiffness curve is depicted in Fig 3(b). In summary, the parameters of the ABR pipe are listed in Table 1. According to the Chinese code CJJ 101–2016 [18], medium sand was selected as the backfill material around the pipe, and the grading curve and mechanical properties are shown in Fig 4 and Table 2.

**Table 1:** The performance parameters of ABR pipe.

Material	Outside diameter (mm)	Internal diameter (mm)	Thickness (mm)	Nominal pressure (MPa)	Yield strength (MPa)	Elasticity modulus (MPa)	Elongation at break
ABR	200	187.4	6.2	1.0	40.07	3012.50	0.60

**Table 2:** Properties of soil.

Soil type	Density (kg/m^3^)	Maximum dry density (kg/m^3^)	Relative density (kg/m^3^)	Poisson’s ratio	Elasticity modulus (MPa)	Cohesive force (kPa)	Internal friction angle	Expansion angle
Medium sand	1.69 × 10^3^	1.77 × 10^3^	95.48%	0.24	14.20	0.1	30°	25°
Clay	1.71 × 10^3^	1.92 × 10^3^	89.06%	0.28	12.51	34.20	28°	15°

### Experimental plans

**Fig 5:**
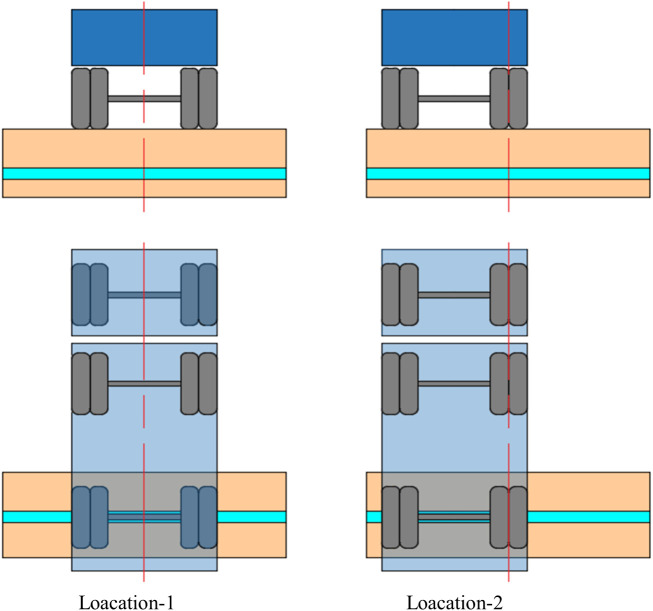
Location of vehicle.

The vehicle load magnitude and wheel-ABR pipe relative position were used as experimental variables for traffic load. According to the Chinese code JTG D60-2015 [21], the maximum limit value for vehicle axle load was 100 kN (i.e., the single axle load should not exceed 10 tons). The vehicle load consisted of two parts: the self-weight of the vehicle and the load. The specific load values were determined on-site for each experiment using a weighbridge. The rear axle load magnitudes of 5 t, 10 t, and 15 t were selected to consider the effect of the overload on the buried ABR pipe. Two relative location relationships between the vehicle and the pipe centerline were designed for the experiments, as shown in Fig 5. Location-1 was where the vehicle axis coincided with the centerline of the pipe, and location-2 was where the right wheel of the vehicle coincided with the centerline of the pipe. The truck was required to follow the specified route for each experiment. The internal pressure ranged from 0 to 1.2 MPa. The loading conditions for the experiment are shown in Table 3.

**Table 3:** Design of experimental loading.

Load	Loading condition					
	1	2	3	4	5	6
Weight of load	5 t		10 t		15 t	
Vehicle location	Location-1	Location-2	Location-1	Location-2	Location-1	Location-2
Internal pressure	0 ~ 1.2 MPa		0 ~ 1.2 MPa		0 ~ 1.2 MPa	

**Fig 6:**
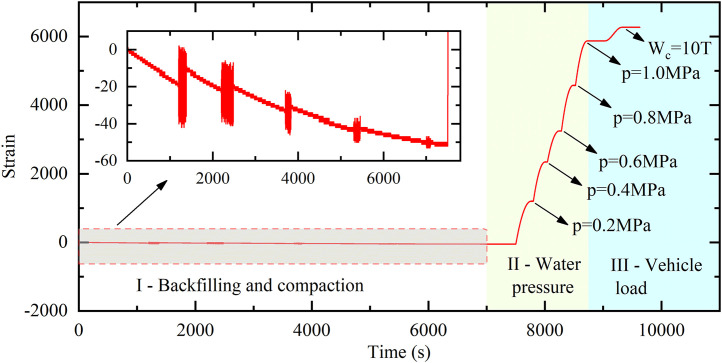
Loading pattern.

A set of circumferential strain data for the whole process of the ABR pipe was selected to illustrate the loading pattern, as depicted in Fig 6. The loading process was divided into four stages: backfill and compaction, internal pressure loading, coupling loading of traffic load and internal pressure, and traffic load loading. The pipe was backfilled and compacted before the experiment loaded. During this stage, circumferential strain increased with the compaction and thickness of the soil. After backfill and compaction, the internal pressure was applied to the pipe. The internal pressure was increased by 0.2 MPa for each loading step to observe the effect of internal pressure changes on the ABR pipe. After each loading step of internal pressure loading was completed, the traffic load was applied to the buried ABR pipe, with the 5 t loading increment. During the test, the vehicle slowly approached the loading position from a distance and turned off its engine to simulate the static load of a vehicle. The loading lasted for 10 minutes, during which data was collected until it stabilized (±2% over 1 min). Subsequently, the vehicle slowly moved away, and its weight distribution was adjusted in preparation for the next loading. The internal pressure was released after it reached 1.2 MPa. Finally, only the traffic load was applied, maintaining the same increment as the previous loading steps until the experiment was completed.

**Fig 7:**
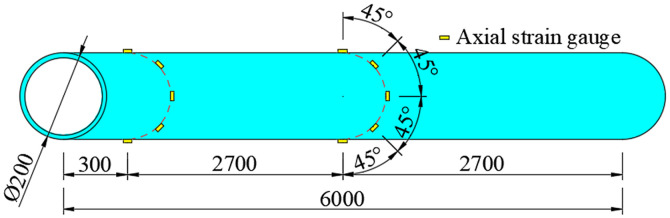
Strain gauges and soil pressure transducer arrangement.

The circumferential strain of the ABR pipe and the soil pressure around the pipe were measured using strain gauges and soil pressure transducers, respectively. The measurement points were arranged as shown in Fig 7. The strain gauges and soil pressure transducers were arranged at the outer walls of the ABR pipe. Both measuring elements were arranged in the direction from the top of the pipe along the circumferential 45° toward the bottom of the pipe.

## Experiment results

### Location-1 loading

**Fig 8:**
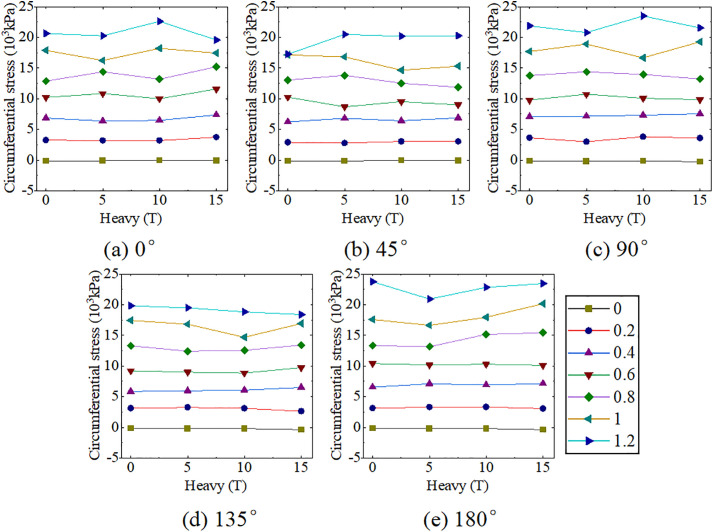
ABR pipe circumferential stress at location-1 loading.

The experiment results of Location-1 loading are presented in Fig 8 and Table 4. When only internal pressure was loaded, the ABR pipe was mainly under tensile stress, and circumferential tensile stress increased with the growth of internal pressure. Under the same internal pressure, the circumferential tensile stress in the ranges of 0° to 90° and 90° to 180° initially decreased and then increased. The most unfavorable pressure and angle under internal pressure were 1.2 MPa and 180°, respectively, and the peak tensile stress was 23751.25 kPa. When only traffic load was applied, the ABR pipe experienced compressive stress. The horizontal distance between the measurement area and the vehicle wheel was 0.6 m. No direct load was applied to the upper part of the pipe, and the horizontal distance exceeded the load diffusion distance [21]. Hence, the increased traffic load slightly impacted the mid-section compressive stress of the ABR pipe. The circumferential compressive stress in the pipe at different angles increased with the growth of traffic load. The most unfavorable conditions under traffic load were 15 t and 180°, and the peak compressive stress was −338.84 kPa. The influence of internal pressure on the circumferential stress of the ABR pipe was more significant than that of traffic load when the traffic load and internal pressure coupling action. Only 0.2 MPa internal pressure counteracted the compressive stresses generated by traffic loads and backfill soil, after which the pipe was only subjected to tensile stress. Hence, under the traffic load and internal pressure coupling action, the most unfavorable conditions for the ABR pipe were 1.2 MPa, 15 t, and 180°, with a peak tensile stress of 23465.04 kPa.

**Table 4:** Location-1 loading circumferential stress experiment results.

Angel	Traffic load (t)	Internal pressure (MPa)						
		0	0.2	0.4	0.6	0.8	1.0	1.2
		Circumferential stress (kPa)						
0° 0° 0° 0°	0	−174.04	3238.42	6840.28	10221.07	12859.37	17898.52	20630.11
	5	−126.68	3188.46	6362.26	10861.05	14389.44	16241.76	20265.54
	10	−47.15	3196.25	6482.77	10002.20	13184.01	18224.45	22608.28
	15	−109.90	3733.64	7372.66	11580.71	15208.40	17449.92	19558.14
45° 45° 45° 45°	0	−155.37	2880.75	6204.63	10259.03	13051.08	17153.76	17260.85
	5	−143.29	2769.76	6800.54	8695.03	13810.76	16807.75	20485.37
	10	−46.81	3030.47	6406.13	9540.06	12541.64	14674.28	20207.33
	15	−110.99	3016.04	6867.80	8992.20	11887.77	15303.24	20259.99
90° 90° 90° 90°	0	−152.88	3615.96	7056.82	9786.01	13792.47	17689.89	21892.34
	5	−205.22	2995.27	7152.40	10739.16	14411.75	18893.96	20799.89
	10	−146.44	3784.21	7329.61	10080.02	13976.17	16665.43	23495.32
	15	−263.95	3573.37	7544.74	9844.69	13240.52	19277.52	21536.44
135° 135° 135° 135°	0	−147.15	3088.93	5856.17	9238.98	13307.14	17436.48	19837.79
	5	−193.20	3263.82	5967.52	9006.13	12417.07	16811.52	19482.61
	10	−183.15	3114.01	6067.87	8867.03	12584.01	14682.57	18833.78
	15	−382.80	2645.30	6526.20	9757.78	13421.44	16941.03	18401.37
180°	0	−157.13	3155.70	6558.89	10403.05	13346.97	17585.66	23751.25
180°	5	−204.18	3287.30	7104.60	10197.36	13151.91	16634.03	20936.76
180°	10	−203.88	3311.41	6908.84	10296.73	15184.08	17969.03	22832.22
180°	15	−388.84	3083.28	7146.72	10114.49	15444.18	20153.47	23465.04

### Location-2 loading

**Fig 9:**
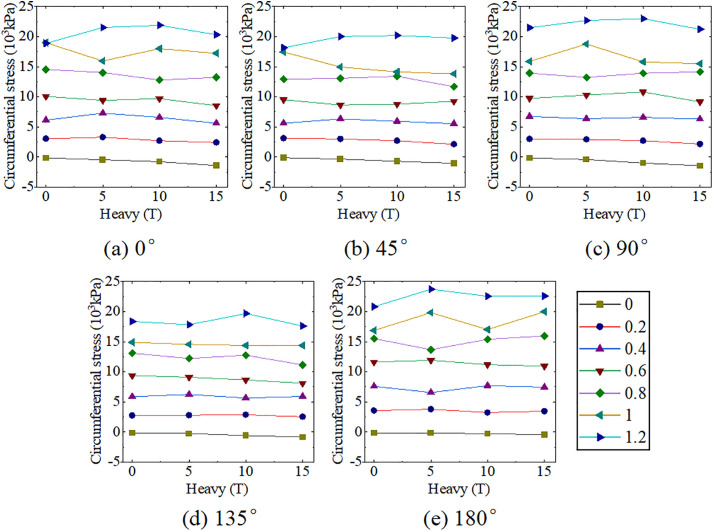
ABR pipe circumferential stress at location-2 loading.

The experiment results under location-2 loading are presented in Fig 9 and Table 5. When only internal pressure was applied, the behavior was consistent with that observed in location-1 loading. When subjected to traffic load alone, the ABR pipe experienced compressive stress. The circumferential stress at different angles within the pipe increased with the growth of the traffic load. The most unfavorable conditions under traffic loading were 15 t and 90°, with a peak pressure of −1458.88 kPa. The peak circumferential compressive stress of location-2 loading increased by 275.19% compared to location-1 loading. Different from the location-1 loading, circumferential tensile stress increased with the angle in the range of 0° to 90°, while the circumferential tensile stress decreased with the angle in the range of 90° to 180°. The reason was that internal pressure was the primary factor affecting the stress distribution of the pipe when the two loads were coupling. The external surface stress on the ABR pipe exhibited uniform tensile stress under internal pressure. However, the external surface stress on the ABR pipe was reduced due to the soil pressure. Under traffic load-internal pressure coupling action, increased traffic load significantly reduced circumferential tensile stress when internal pressure was below 0.6 MPa. Afterward, the effect of traffic load on circumferential stress in the ABR pipe was significantly decreased as the internal pressure increased. In summary, the most unfavorable conditions under traffic load-internal pressure coupling action were 15 t, 1.2 MPa, and 180°, with a maximum peak circumferential tensile stress of 22585.79 kPa. Compared to location-1 loading, the maximum peak stress differed by only 4%, highlighting the significant influence of internal pressure on the circumferential stress in the ABR pipe compared to the traffic load. Notably, under traffic load-internal pressure coupling, the circumferential stress in the ABR pipe differed by only 28.39 kPa at 10 t and 15 t, which was attributed to soil plasticity. Hence, the state of backfilled soil should be considered when considering the most unfavorable conditions under traffic load-internal pressure coupling action.

**Table 5:** Location-2 loading circumferential stress experiment results.

Angel	Traffic load (t)	Internal pressure (MPa)						
		0	0.2	0.4	0.6	0.8	1.0	1.2
		Circumferential stress (kPa)						
0°	0	−156.80	3022.19	6102.70	10060.26	14548.86	18967.54	18889.40
0°	5	−455.42	3301.37	7293.19	9417.35	14033.66	15936.82	21509.67
0°	10	−785.17	2694.46	6597.82	9707.69	12786.91	17987.26	21870.93
0°	15	−1399.65	2415.76	5615.48	8522.34	13219.30	17242.12	20319.51
45°	0	−137.76	3091.57	5584.07	9516.85	12934.40	17446.27	18125.74
45°	5	−351.20	3007.54	6333.61	8647.47	13078.51	14961.79	20011.34
45°	10	−702.49	2685.83	5925.88	8808.03	13397.28	14144.83	20204.49
45°	15	−1054.28	2102.55	5508.32	9273.03	11685.85	13832.39	19757.73
90°	0	−174.57	2982.24	6718.91	9743.13	13939.97	15886.04	21472.89
90°	5	−414.61	2938.73	6371.66	10284.95	13210.26	18759.38	22674.01
90°	10	−992.77	2694.23	6594.00	10790.86	13926.98	15811.48	22989.34
90°	15	−1458.88	2164.20	6292.18	9165.88	14138.77	15487.60	21215.45
135°	0	−141.11	2721.13	5842.68	9367.69	13095.88	14871.99	18368.29
135°	5	−270.16	2780.08	6237.66	9078.86	12206.14	14548.20	17812.88
135°	10	−587.81	2847.11	5620.92	8643.20	12745.55	14361.03	19685.27
135°	15	−858.86	2536.22	5899.37	8109.23	11134.67	14360.56	17609.10
180°	0	−156.46	3528.29	7587.25	11608.92	15490.39	16865.06	20807.12
180°	5	−179.99	3761.03	6568.28	11922.77	13684.57	19817.10	23698.25
180°	10	−308.01	3223.83	7674.01	11170.53	15386.57	17004.65	22557.40
180°	15	−466.94	3453.26	7442.80	10900.29	15930.84	19971.33	22585.79

In summary, circumferential compressive and tensile stresses in the buried ABR pipe were caused by traffic load and internal pressure, respectively. Traffic load affected circumferential compressive stress directly beneath the ABR pipe, and the influence decreased significantly as the horizontal distance increased. The most unfavorable loading conditions and circumferential stresses for location-1 and location-2 are presented in Table 6. The maximum internal pressure and design value for traffic load for the water distribution pipeline were 1.0 MPa and 10 t, respectively. Hence, the finite element extended analysis was performed with 1.0 MPa and 10 t as the limit state.

**Table 6:** The most unfavorable conditions and circumferential stress of experiments.

Loading location	Loading combination	Circumferential angle	Traffic load (t)	Internal pressure (MPa)	Experiment result (kPa)
Location-1	Internal pressure	180°	0	1.2	23751.25
	Traffic load	180°	15	0	−388.84
	Coupling action	180°	15	1.2	23465.04
Location-2	Internal pressure	180°	0	1.2	23751.25
	Traffic load	90°	15	0	−1458.88
	Coupling action	180°	15	1.2	22585.79

## Numerical methods

Previously, experimental experiments and analyses were conducted on buried ABR pipelines under vehicle loads. However, due to the limited number of experiment conditions and the singularity of the experiment results, a numerical analysis study will be carried out on the experiments to facilitate a more comprehensive analysis.

### Finite element modeling

**Fig 10:**
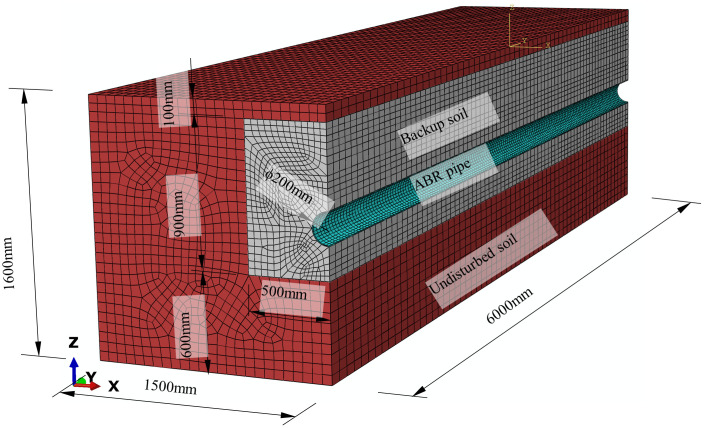
Geometric model and meshing of buried ABR pipe.

The geometric model and meshing are presented in Fig 10. Finite element simulations of buried ABR pipes under the traffic load-internal pressure coupling effect were conducted using ABAQUS software. The calculation solid model of the buried ABR pipe was established, where the Y and Z axes represented the axial and gravity direction, respectively. Since the ideal experiment specimen was symmetric about the YOZ plane, the symmetric boundaries were applied during the calculation, and the half model was established. The model consisted of undisturbed soil, backfill soil, and ABR pipe. The dimension of the undisturbed soil was 6000 mm × 1500 mm × 1600 mm (length × width × height), and the dimension of the backfill sand was 6000 mm × 500 mm × 900 mm (length × width × height). The outer diameter, thickness, and length of the ABR pipe were 100 mm, 6.2 mm, and 6000 mm, respectively. The ABR pipe adopted C3D8 elements with the neutral axis algorithm for meshing. Backfill and undisturbed soil utilized hexahedral elements predominantly with the neutral axis algorithm for meshing. The element types included C3D6 and C3D8 elements. The finite element model comprised 87342 nodes and 77040 elements, including 76590 C3D8 elements and 450 C3D6 elements.

**Fig 11:**
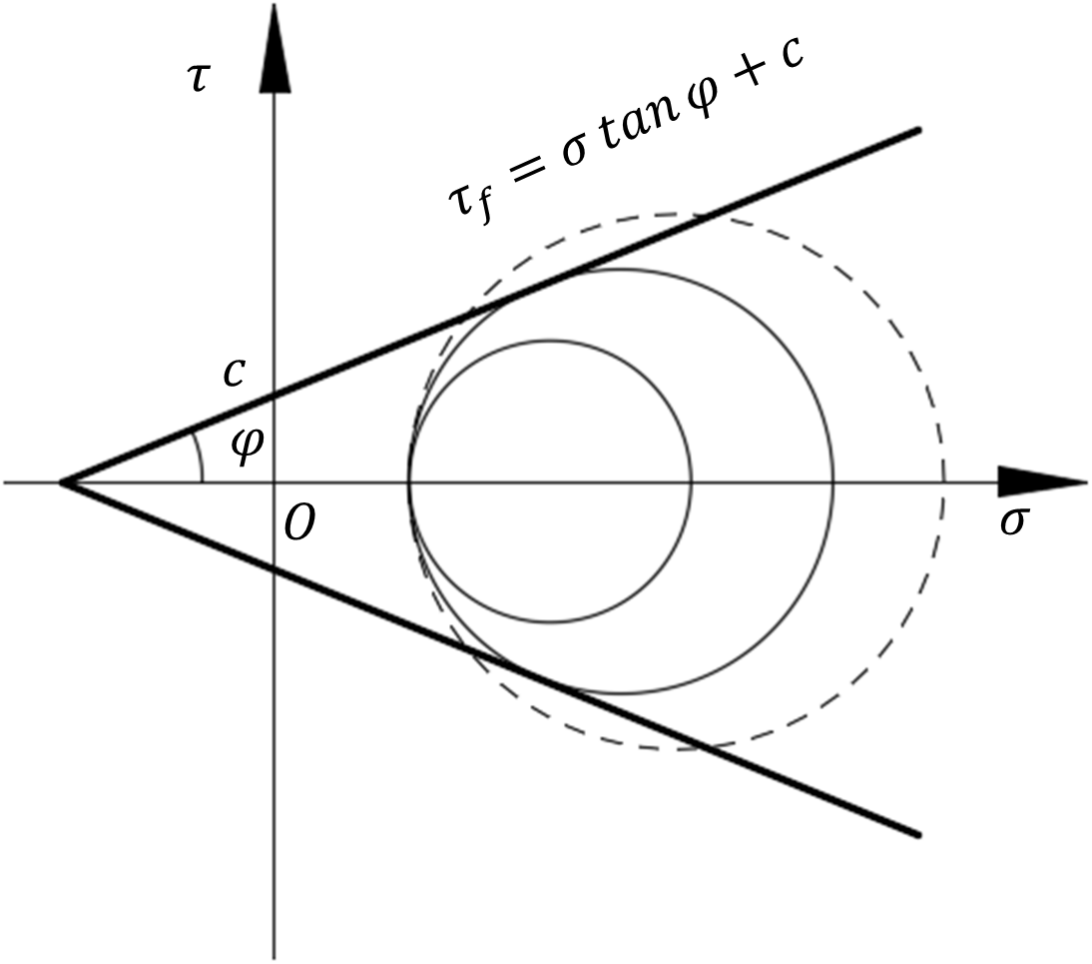
Mohr-Coulomb strength theory model.


The soil parameters used in the numerical simulation in this study are shown in Table 2. As depicted in Fig 11, the soil model used the Mohr-Coulomb strength theory model, which considered that the shear force reaching the shear strength of the soil leads to failure. The theory proposed that the shear strength of sandy soil can be expressed as a linear function of the normal stress on the shearing sliding surface, as shown in the following equation:

$$\tau_{f}=\sigma \;tan\;\varphi$$ (1)

Where $\tau_{f}$ is the soil shear strength; σ is the normal stress; φ is the internal friction angle. For cohesive soils, the modified shear strength equation after considering cohesion c is shown as follows:


$$\tau_{f}=\sigma \;tan\;\varphi +c$$ (2)

**Fig 12:**
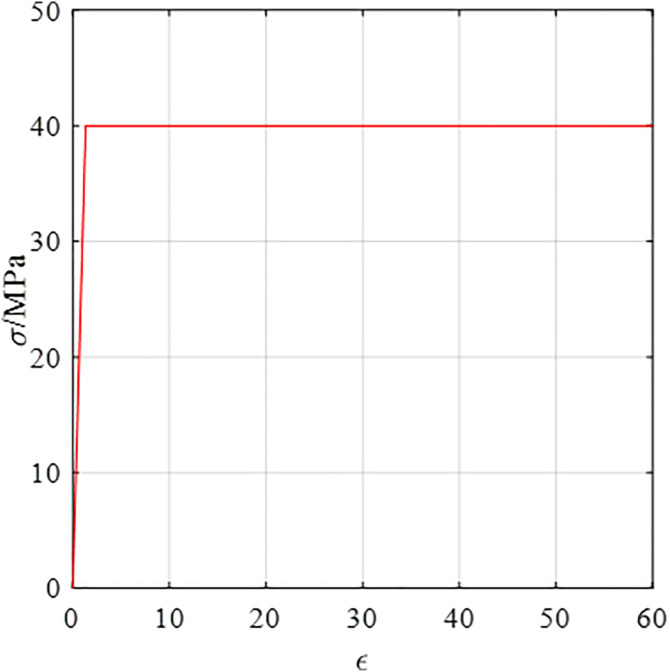
Constitutive curve of ABR pipe material.


Based on the stress-strain curves of ABR pipe material, the general elastic-perfectly plastic constitutive model was adopted. The ABR pipe material adopted the double broken line model, and the constitutive curve is illustrated in Fig 12. The material followed Hooke’s law in the elastic stage and maintained constant stress (i.e., yield stress) until failure after reaching the yield point. The expressions for the elastic stage(i.e., Hooke’s law) and the perfectly plastic stage (i.e., the stress invariant stage) for ABR material are respectively given as follows:

$$\sigma_{\text{c},\text{p}}=E_{\text{p}}\varepsilon_{\text{c},\text{p}}$$ (3)

$$\sigma_{\text{c},\text{p}}=\sigma_{c,py}$$ (4)

$\sigma_{c,p}$ is the $E_{p}$ is the elasticity modulus; $\varepsilon_{c,p}$ is the strain; $\sigma_{c,py}$ is the yield stress; c is the criterion; p is the pipe; y is the yield.

### Boundary condition and interaction

Symmetric boundary conditions were applied on the YOZ plane for all components to reflect the constraints of the buried environment. Fixed boundary conditions were set on the bottom and left surfaces of the undisturbed soil and the front and rear surfaces of all components. The upper boundary of the undisturbed soil was considered the free boundary.

The entire model was subjected to the gravitational acceleration of 9.8 m/s² along the z-axis, and the soil initial stress was simulated based on the soil layer thickness. Pressure loads were applied on the inner surface of the ABR pipe to simulate static water pressure, and the load magnitude correlated to experiments. The vehicular load was applied on a 100 mm × 600 mm region on the upper surface of the undisturbed soil directly above the pipe. A region of 100 mm × 600 mm was defined on the upper surface of the undisturbed soil directly above the pipe, where the vehicular load was applied with the load magnitude determined by axle weight. The backfill soil was compacted and naturally consolidated, forming a tight bond with the undisturbed soil. Hence, the contact behavior between undisturbed and backfill soil was simulated using the Tie connection. Surface-to-surface contact was adopted to interact with the backfill soil and the ABR pipe. Hard contact was used in the normal direction, and frictional contact was used in the tangential direction with a friction coefficient of 0.3.

### Finite element model verification

**Fig 13:**
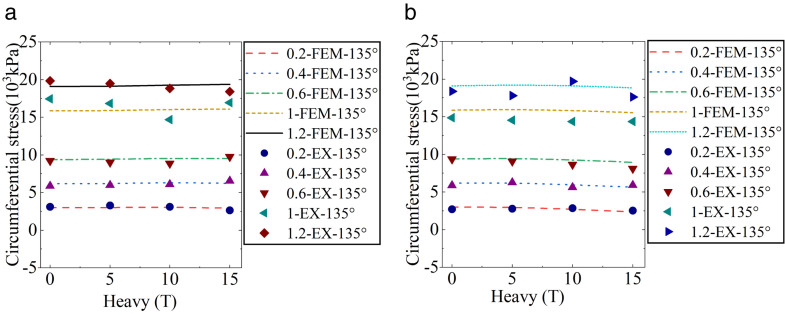
Error analysis. (a) Location-1 loading. (b) Location-2 loading.

The comparison of test results and the error between finite element simulations and test results are presented in Tables 7 and 8, respectively. The average and error values of the tests and finite element simulations were small, indicating the accuracy of the finite element simulations. Representative curves for different loading locations were selected to investigate the errors specifically. The representative curves of location 1 loading are shown in Fig 13(a). The most accurate simulation was achieved at the internal pressure of 0.2 MPa and the load of 10 t, with an error of 1.45%. The maximum deviation occurred at the internal pressure of 0.2 MPa and the load of 15 t, with an error of 11.08%. Location 2 loading is shown in Fig 13(b). The simulation was most accurate at the internal pressure of 0.6 MPa and the load of 0, with an error of 0.14%. The maximum deviation occurred at the internal pressure of 0.6 MPa and the load of 15 t, with an error of 10.29%. It was worth noting that the curves not mentioned were visually biased, but the actual calculated errors were relatively small.

**Table 7:** Finite element simulation results.

Angel	Internal pressure (MPa)	Location-1				Location-2		
		Traffic load (t)						
		0	5	10	15	5	10	15
		Circumferential stress (kPa)						
0°	0	−158.52	−138.80	−44.74	−109.43	−429.58	−844.97	−1299.93
0°	0.2	3250.23	3315.50	3520.15	3476.26	3138.58	2738.91	2284.95
0°	0.4	6723.31	6812.20	7062.94	7065.57	6705.15	6320.14	5870.19
0°	0.6	10224.20	10325.70	10592.40	10648.20	10267.40	9900.64	9456.25
0°	0.8	13738.40	13846.50	14111.70	14226.20	13825.20	13477.50	13044.70
0°	1.0	17260.60	17372.70	17625.00	17799.30	17383.50	17060.00	16629.70
0°	1.2	20789.20	20902.20	21144.70	21371.00	20937.70	20637.10	20216.50
45°	0	−148.22	−132.64	−44.99	−118.66	−385.09	−757.65	−1160.95
45°	0.2	2990.64	3031.82	3226.50	3179.77	2882.91	2539.39	2136.92
45°	0.4	6182.11	6236.80	6481.03	6473.83	6147.55	5835.32	5435.01
45°	0.6	9407.18	9467.20	9723.62	9768.90	9419.64	9125.06	8730.82
45°	0.8	12648.50	12709.70	12956.60	13062.30	12687.10	12412.30	12027.70
45°	1.0	15899.50	15960.40	16184.90	16350.70	15957.10	15697.70	15323.80
45°	1.2	19158.20	19216.60	19421.20	19636.20	19223.80	18983.10	18624.10
90°	0	−169.01	−190.45	−151.50	−284.14	−449.07	−920.84	−1471.14
90°	0.2	3299.26	3307.75	3474.51	3380.54	3155.79	2711.74	2165.56
90°	0.4	6820.03	6842.55	7073.02	7035.31	6754.46	6344.40	5804.12
90°	0.6	10380.30	10408.40	10656.50	10682.80	10360.10	9978.50	9456.60
90°	0.8	13963.70	13992.10	14232.90	14331.10	13962.60	13620.90	13110.80
90°	1.0	17562.10	17588.60	17810.30	17972.20	17572.60	17263.30	16765.60
90°	1.2	21171.10	21192.90	21396.20	21610.50	21184.10	20905.00	20424.30
135°	0	−149.94	−190.54	−202.68	−358.79	−277.75	−575.81	−913.66
135°	0.2	2982.16	2979.39	3068.71	2938.27	2976.07	2702.33	2368.88
135°	0.4	6165.03	6180.89	6322.00	6229.20	6222.62	5980.04	5653.07
135°	0.6	9381.21	9403.03	9565.76	9514.69	9472.06	9261.53	8943.82
135°	0.8	12612.30	12634.60	12801.00	12800.70	12717.70	12546.70	12236.10
135°	1.0	15853.70	15874.90	16033.20	16082.60	15969.50	15830.50	15532.20
135°	1.2	19101.60	19121.10	19268.50	19362.00	19221.50	19114.30	18832.90
180°	0	−161.59	−202.82	−212.09	−367.43	−183.39	−282.39	−437.27
180°	0.2	3405.09	3419.70	3513.90	3385.74	3517.55	3455.05	3308.24
180°	0.4	7037.65	7077.24	7221.39	7134.73	7218.15	7191.27	7054.29
180°	0.6	10701.20	10751.30	10917.30	10880.40	10917.70	10927.90	10805.70
180°	0.8	14378.50	14431.50	14605.60	14619.80	14613.40	14661.00	14556.60
180°	1.0	18063.80	18116.30	18288.30	18352.50	18312.40	18396.40	18310.40
180°	1.2	21754.60	21805.30	21972.70	22081.90	22009.50	22131.30	22067.10

**Table 8:** Comparison of finite element simulation and experiment results.

Angel	Internal pressure (MPa)	Location-1				Location-2			
		Traffic load (t)							
		0	5	10	15	0	5	10	15
		Error analysis							
0°	0	8.92%	9.57%	5.12%	0.43%	1.09%	5.67%	7.62%	7.12%
0°	0.2	0.36%	3.98%	10.13%	6.89%	7.55%	4.93%	1.65%	5.42%
0°	0.4	1.71%	7.07%	8.95%	4.17%	10.17%	8.06%	4.21%	4.54%
0°	0.6	0.03%	4.93%	5.90%	8.05%	1.63%	9.03%	1.99%	10.96%
0°	0.8	6.84%	3.77%	7.04%	6.46%	5.57%	1.49%	5.40%	1.32%
0°	1.0	3.56%	6.96%	3.29%	2.00%	9.00%	9.08%	5.16%	3.55%
0°	1.2	0.77%	3.14%	6.47%	9.27%	10.06%	2.66%	5.64%	0.51%
45°	0	4.60%	7.43%	3.89%	6.90%	7.59%	9.65%	7.85%	10.12%
45°	0.2	3.81%	9.46%	6.47%	5.43%	3.26%	4.14%	5.45%	1.63%
45°	0.4	0.36%	8.29%	1.17%	5.74%	10.71%	2.94%	1.53%	1.33%
45°	0.6	8.30%	8.88%	1.92%	8.64%	1.15%	8.93%	3.60%	5.85%
45°	0.8	3.08%	7.97%	3.31%	9.88%	2.21%	2.99%	7.35%	2.93%
45°	1.0	7.31%	5.04%	10.29%	6.84%	8.87%	6.65%	10.98%	10.78%
45°	1.2	10.99%	6.19%	3.89%	3.08%	5.70%	3.94%	6.05%	5.74%
90°	0	10.55%	7.20%	3.45%	7.65%	3.19%	8.31%	7.25%	0.84%
90°	0.2	8.76%	10.43%	8.18%	5.40%	10.63%	7.39%	0.65%	0.06%
90°	0.4	3.36%	4.33%	3.50%	6.75%	1.51%	6.01%	3.79%	7.76%
90°	0.6	6.07%	3.08%	5.72%	8.51%	6.54%	0.73%	7.53%	3.17%
90°	0.8	1.24%	2.91%	1.84%	8.24%	0.17%	5.70%	2.20%	7.27%
90°	1.0	0.72%	6.91%	6.87%	6.77%	10.55%	6.33%	9.18%	8.25%
90°	1.2	3.29%	1.89%	8.93%	0.34%	1.41%	6.57%	9.07%	3.73%
135°	0	1.90%	1.38%	10.66%	6.27%	6.26%	2.81%	2.04%	6.38%
135°	0.2	3.46%	8.71%	1.45%	11.08%	9.59%	7.05%	5.09%	6.60%
135°	0.4	5.27%	3.58%	4.19%	4.55%	5.52%	0.24%	6.39%	4.18%
135°	0.6	1.54%	4.41%	7.88%	2.49%	0.14%	4.33%	7.15%	10.29%
135°	0.8	5.22%	1.75%	1.72%	4.63%	3.69%	4.19%	1.56%	9.89%
135°	1.0	9.08%	5.57%	9.20%	5.07%	6.60%	9.77%	10.23%	8.16%
135°	1.2	3.71%	1.86%	2.31%	5.22%	3.99%	7.91%	2.90%	6.95%
180°	0	2.84%	0.67%	4.03%	5.51%	3.28%	1.89%	8.32%	6.35%
180°	0.2	7.90%	4.03%	6.11%	9.81%	3.49%	6.47%	7.17%	4.20%
180°	0.4	7.30%	0.39%	4.52%	0.17%	7.24%	9.89%	6.29%	5.22%
180°	0.6	2.87%	5.43%	6.03%	7.57%	7.82%	8.43%	2.17%	0.87%
180°	0.8	7.73%	9.73%	3.81%	5.34%	7.18%	6.79%	4.72%	8.63%
180°	1.0	2.72%	8.91%	1.78%	8.94%	7.11%	7.59%	8.18%	8.32%
180°	1.2	8.41%	4.15%	3.76%	5.89%	4.55%	7.13%	1.89%	2.30%
Average value					5.35%				5.57%
Standard deviation					2.89%				2.98%

## Numerical results

The study investigated the most unfavorable conditions affecting the circumferential maximum tensile and compressive stress in buried ABR pipes through experiments and finite element simulations. It was necessary to determine the axial most unfavorable location and the diameter-thickness ratio to extend the analysis of the mechanical performance of buried ABR pipe under the traffic load-internal pressure coupling action.

### The most unfavorable location in the axial direction

**Fig 14:**
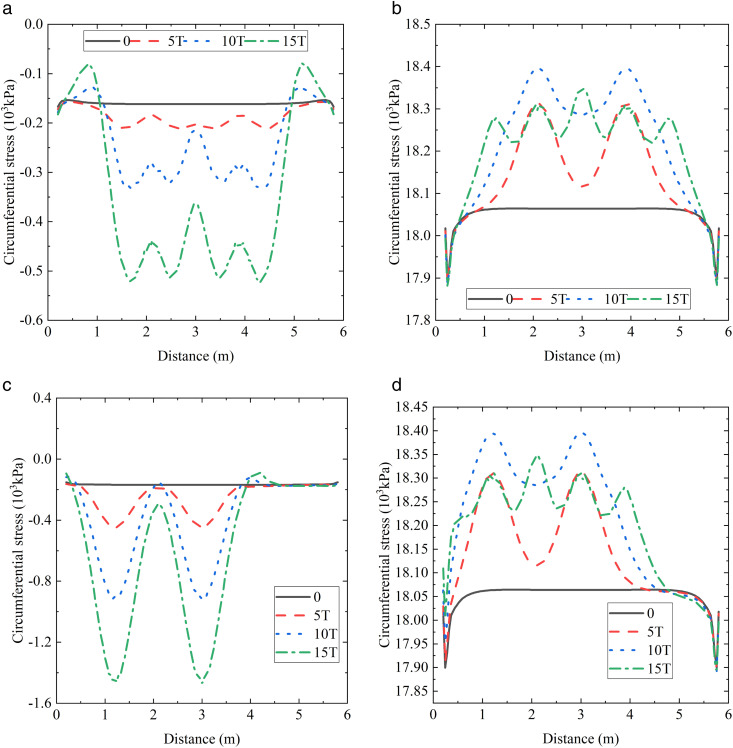
ABR pipe axial stress. (a) 0 MPa, 180° and location-1. (b) 1.0 MPa, 180°and location-1. (c) 0 MPa, 90° and location-2. (d) 1.0 MPa, 180° and location-2.

The experiment and finite element simulation results indicated that the most unfavorable angle for the ABR pipe was 180° at location-1. The circumferential stress distribution along the axial direction of the buried ABR pipe at 180° is shown in Fig 14(a–b). Under traffic loads, the maximum axial value of circumferential stress was only −523.63 kPa. Consequently, only the separate internal pressure and coupling action were considered. Traffic load was applied above 1.8 m ~ 2.4 m and 3.6 m ~ 4.2 in the axial direction of the ABR pipe. The most unfavorable axial locations were 2.1 m and 3.9 m. The two locations were at the center of the traffic load, and the circumferential stress was equal.

For location-2 loading, the most unfavorable angles for compressive and tensile stresses were 90° and 180°, respectively. The circumferential stress distribution along the axial direction is presented in Fig 14(c–d). Traffic load was applied above 0.9 m ~ 1.5 m and 2.7 m ~ 3.3 m in the axial direction of the ABR pipe. The most unfavorable axial locations were at 1.2 m and 3.0 m in the centers of the traffic load, and the circumferential stress at the two locations was equal.

In summary, the most unfavorable axial locations were located directly beneath the traffic load center, and the influence of circumferential stress was minor when only traffic load was applied.

### Effect of ABR pipe diameter-thickness ratio

**Fig 15:**
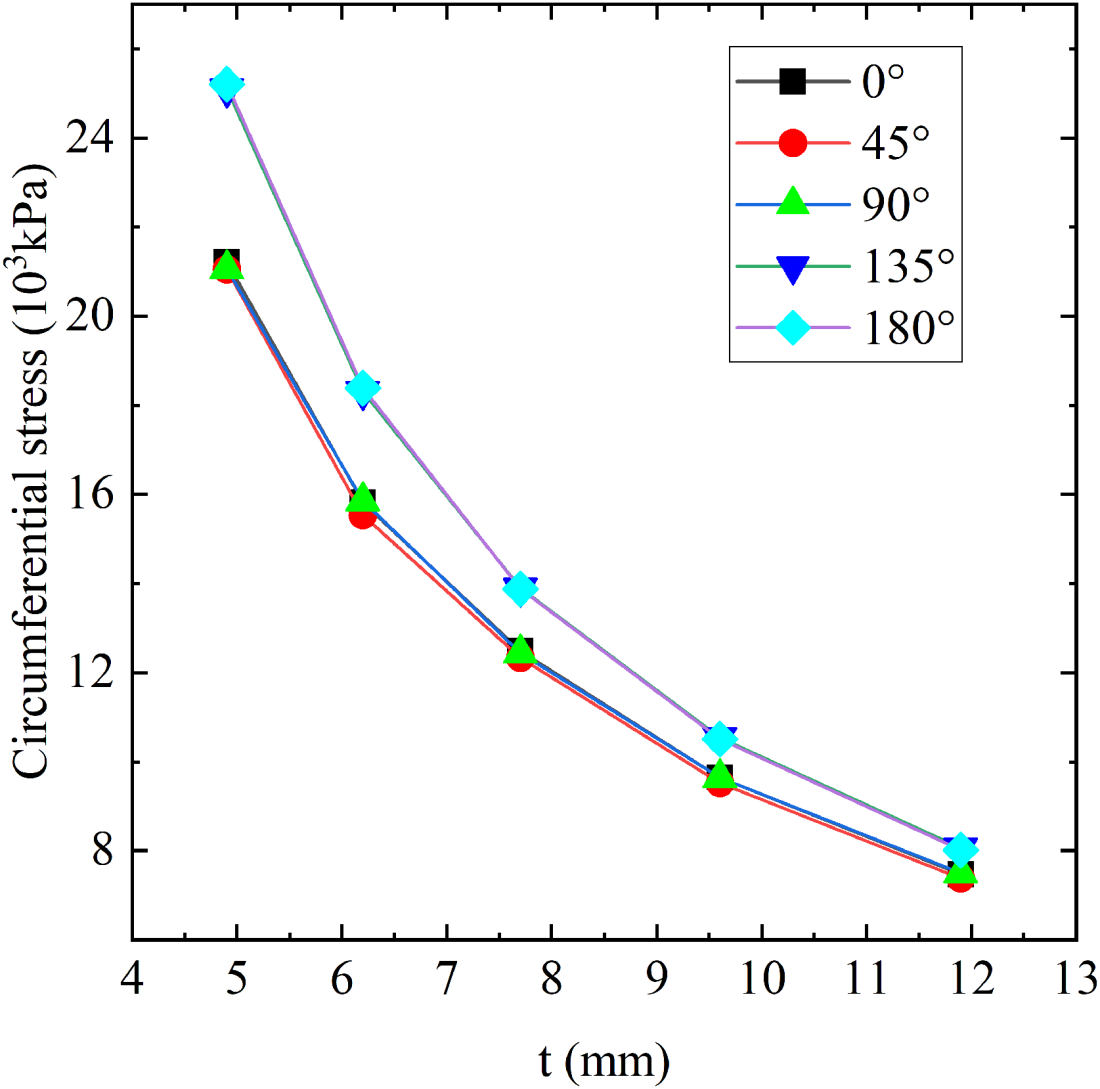
Circumferential stress in ABR pipe with different thicknesses. (a) 5 **t.** (b) 10 **t.** (c) 15 t.

The circumferential stress for ABR pipes of different thicknesses and the diameter-to-thickness ratio and thickness of ABR pipes are shown in Fig 15 and Table 9, respectively. Based on the above unfavorable factors, finite element comparison analysis was conducted for ABR pipes with various thicknesses (4.9 mm to 11.9 mm). With the wall thickness increased (i.e., the diameter-to-thickness ratio was decreased), the tensile stress in the ABR pipe was significantly decreased. The increased wall thickness increased cross-sectional area and circumferential stiffness, enhancing the bearing capacity of ABR pipe. Wall thickness significantly affected 180° circumferential tensile stress, with a decrease of 68.17%. The rest of the angle circumferential stress change pattern was consistent. Considering the practical application for water distribution pipes, the wall thickness of 6.2 mm was chosen for further analysis.

**Table 9:** ABR pipe diameter-thickness ratio.

Wall thickness (mm)	4.9	6.2	7.7	9.6	11.9
Diameter-thickness ratio	40.82	32.26	25.97	20.83	16.81

### Traffic load-internal pressure coupling action

**Fig 16:**
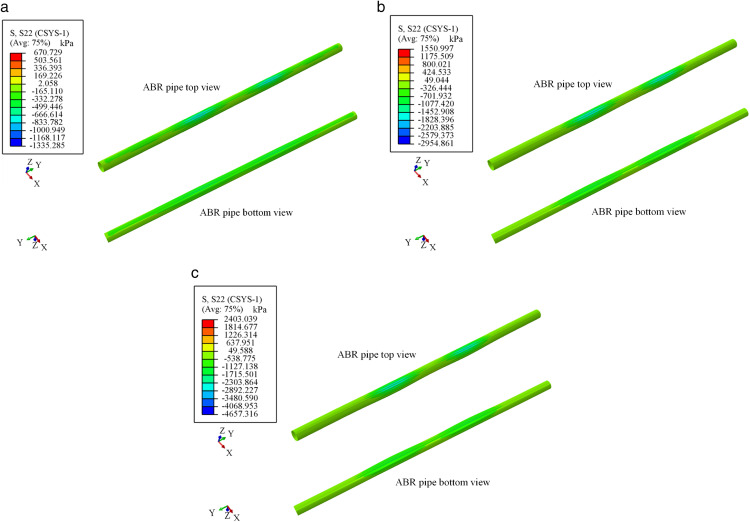
Stress nephogram of ABR pipe at location-1 loading.

**Fig 17:**
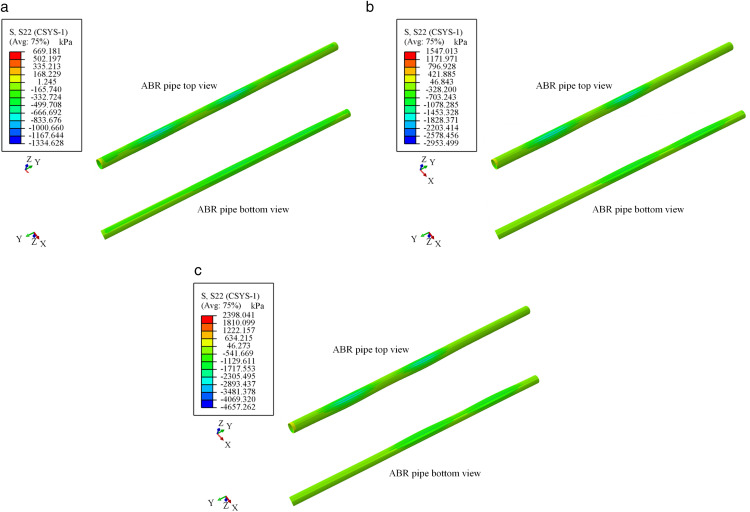
Stress nephogram of ABR pipe at location-2 loading. (a) 5 **t.** (b) 10 **t.** (c) 15 t.

The circumferential stress nephogram of ABR pipes under traffic loads is illustrated in Figs 16 and 17. The stress nephogram deformation coefficient was magnified tenfold. Under traffic load, the ABR pipe sustained compressive stress, and noticeable deformation was observed within approximately 0.3 m range on either side of the wheel. The effect range of traffic load on the ABR pipe was approximately 0.3 m, and beyond this range, the influence of traffic load can be neglected. The circumferential stress at the bottom of the pipe was higher than that at the top, with the maximum circumferential stress occurring directly beneath the wheel center.

**Fig 18:**
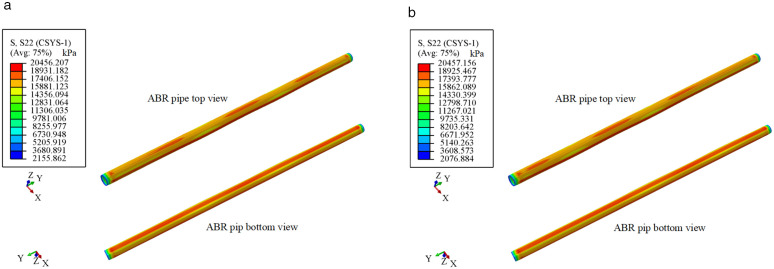
Circumferential stress nephogram of ABR pipe. **(a)** Location-1. **(b)** Location-2.

As shown in Fig 18, to validate the effectiveness of the mechanical performance of buried ABR pipes under the traffic load-internal pressure coupling action, circumferential stress nephogram were analyzed for two different loading locations under conditions of 10 t traffic load, 1.0 MPa internal pressure, and 6.2 mm wall thickness. The stress variation pattern of ABR pipes was similar under the two different loading locations. It can be observed that stress concentration occurred directly beneath the wheel center at the top of the ABR pipe, and the circumferential stress in the axial direction was rapidly decreased with the increased horizontal distance of the traffic load. However, the stress at the bottom of the pipe was uniformly distributed, indicating the dominant influence of internal pressure over traffic loads. Additionally, the deformation of the ABR pipe at the bottom was slight compared to the top. The reason was that the resistance provided by internal pressure against the effect of traffic load prevented the transmission of traffic loads from the top to the bottom. Therefore, the mechanical performance of buried ABR pipe was primarily influenced by internal pressure.

## Calculation model of ABR pipe circumferential stress

The stress on the buried ABR pipe under traffic load was due to the transfer of soil pressure generated by external loads and soil self-weight to the pipe through the backfill soil around the pipe. Hence, the calculation of vertical soil pressure on the pipe was the foundation for studying the mechanical performance of buried ABR pipes. Currently, the calculation of vertical soil pressure on buried flexible pipes under traffic loading is primarily based on Marston’s load theory [22] and the prism load method [23]. When the aspect ratio of the trench was typical, the results of the two methods were close and could be selected reasonably according to specific circumstances. When the pipe was filled with water, the circumferential stress generated by internal pressure was opposite to that generated by traffic load. The circumferential stress in the buried ABR pipe under internal pressure can be calculated according to the theory of thin shells. In summary, the calculation of circumferential stress of buried ABR pipes under traffic loading-internal pressure coupling action is as follows:


$$\sigma_{ABR}=\sigma_{w}-\sigma_{c,s}-\sigma_{c,t}$$ (5)

Where $\sigma_{ABR}$ is the ABR pipe circumferential stress under traffic load-internal pressure coupling action; $\sigma_{w}$, $\sigma_{c,s}$, and $\sigma_{c,t}$ are the circumferential stress in the ABR pipe due to internal pressure, backfill soil, and traffic load, respectively.

### Calculation of vertical soil pressure

Marston proposed that the soil pressure at the pipe crown is generated by the self-weight of the backfill soil ($W_{t})$, and the soil pressure is uniformly distributed along the entire trench width (B). Hence, the width range is the entire trench when calculating the self-weight of the backfill soil. During the backfill construction of pipes, relative displacement occurred between the backfill soil and the trench wall as the thickness of the backfill soil increased, leading to friction on both sides. The soil pressure at the pipe crown was equal to the self-weight of the soil from the pipe crown to the surface of the backfill soil minus the friction of the trench walls on both sides:


$$\sigma_{v,s}=W_{t}/B=C_{t}\gamma_{s}B$$ (6)

$$C_{t}=(\text{1}-e^{-\text{2}K\mu H/B)}/(\text{2}K\mu )$$ (7)

Where $\sigma_{v,s}$ is calculating vertical soil pressure for Marston's load method; $W_{t}$ is the vertical soil pressure at the pipe crown; K is the lateral soil pressure coefficient; μ is the friction coefficient; H is the depth of overburden above the pipe crown; $\gamma_{s}$ is the gravity of soil. The prism load method assumes that the vertical soil pressure at the pipe crown is equal to the self-weight of the backfill soil above the pipe crown. In contrast to Marston’s load theory, the prism load method calculates the self-weight of the backfill soil within the width of the pipe diameter. According to the Chinese code CECS122−2001 [23] and GB50332−2002 [6], the calculation of the vertical load at the pipe crown using the prism load method is expressed by the following equation:


$$\sigma_{v,s}=\gamma_{s}H$$ (8)

**Fig 19:**
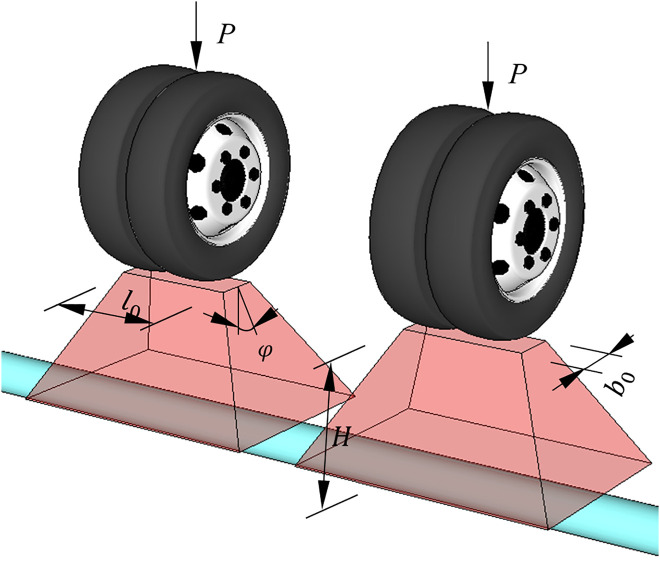
Effects of traffic loading on buried ABR pipe.


As shown in Fig 19, the influence of traffic load on buried ABR pipes is comprehensively considered by converting the traffic loads into vertical soil pressure applied to the pipe crown. When traffic load is converted into vertical soil pressure, the usual practice is to neglect the pipe-soil interaction, leading to the calculated values that are usually conservative. The vertical soil pressure load on the pipe under the traffic loads is calculated using the following formula:

$$\sigma_{v,t}=\frac{P}{A_{\text{H}}}$$ (9)

$$A_{\text{H}}=(l_{\text{0}}+mH)(b_{\text{0}}+mH)$$ (10)

Where $\sigma_{v,s}$ is the vertical earth pressure calculated by the prism load method; P is the unilateral wheel load; $A_{\text{H}}$ is the area of the traffic load spreading to the pipe crown; $l_{\text{0}}$ is the wheel touchdown length;

$b_{\text{0}}$ is the wheel touchdown length width; φ is the spread angle; m is the area spreading coefficient, which takes the value of 2tanφ as recommended by code AASHTO [24,25] and AS/NZS 2566 [25].

### Pipe stress calculation

The relative stiffness coefficient of buried flexible pipes is small, and the bending moment can be neglected when the sectional stress of buried flexible pipes is calculated. Hence, based on the thin-walled assumption, the stress calculation for buried ABR pipes is expressed by the following equation:


$$\sigma_{a}=N/A_{p}$$ (11)

$$\sigma_{a}=\sigma_{c,s}+\sigma_{c,t}$$ (12)

Where $\sigma_{a}$ is the pipe circumferential stress generated by traffic load and soil pressure; $A_{p}$ is the cross-sectional area of the pipe per unit length; N is the circumferential force at any location in the cross-section per unit length.

According to Moore's method [26], the circumferential force generated by traffic load and soil pressure is maximum at the pipe invert (i.e., 90° location). Additionally, the influence of pipe-soil interaction on circumferential force is considered using the complete bonding assumption and neglecting the impact of pipe-soil interaction with the smooth assumption. The circumferential force $N_{sp}$ generated by traffic load and soil pressure at the pipe invert under the complete bonding and smooth assumption is calculated by the following equations:


$$N_{sp}=(\sigma_{v,s}+\sigma_{v,t})R[(\text{1}-\nu_{s})/(\text{3}-\text{2}\nu_{s})][\text{5}-\text{2}\nu_{s}+K(\text{1}-\nu_{s})]$$ (13)

$$N_{sp}=(\sigma_{v,s}+\sigma_{v,t})R(\text{1}-\nu_{s})(\text{1}+K)$$ (14)

Where R is the radius of the pipe and is taken as 1/2 of the nominal diameter; $v_{s}$ and $E_{s}$ are Poisson's ratio and elasticity modulus of the soil, respectively; $E_{p}$ is the elasticity modulus of the ABR pipe; $A_{p}$ is the cross-sectional area per unit length of the pipe; $I_{p}$ is the moment of inertia per unit length of the cross-section.

When only internal pressure was applied to the buried ABR pipe, the stress $\sigma_{w}$ in the pipe cross-section is calculated by the following equation:


$$\sigma_{w}=\frac{\sigma_{e}R}{A_{\text{P}}}$$ (15)

Where $\sigma_{e}$ is the static water pressure inside the pipe.

### Comparison and analysis of calculation results

**Fig 20:**
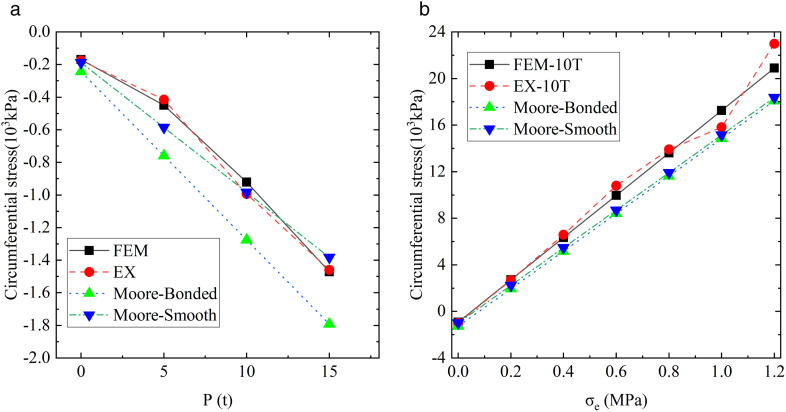
Location-2 loading ABR pipe circumferential stress. **(a)** Traffic load. **(b)** Traffic load-internal pressure.

The position at the pipe invert (i.e., 90° location) and traffic load of 10 t were taken as an example, and the prism load method was employed to calculate the soil pressure above the pipe crown. Subsequently, the above formulas were applied to calculate the circumferential stress of the buried ABR pipe at location-2. The comparison of experiments, simulations, and theoretical results under the traffic loads is depicted in Fig 20(a), and the comparison under the traffic load-internal pressure coupling action is presented in Fig 20(b).

Under the influence of traffic load, the calculated stress of the pipe was small, and the pipe invert was compressed. The theoretical calculation result was −243.12 kPa under the complete bonding assumption and −187.55 kPa under the smooth assumption, compared to the 169.01 kPa by finite element simulation. The friction was assumed in the finite element simulation, and the calculation results were smaller than those under the bonding assumption and larger than those under the smooth assumption. Guiding engineering design with theoretical methods based on the bonding assumption tended toward safety. Under the traffic load-internal pressure coupling action, both theoretical and finite element calculations observed the transition from compressive to tensile stress at the pipe invert. However, the tensile stress calculated through finite element simulation is higher than that of the two theoretical models, and the error increases with internal pressure. The maximum error was 13.52% when the internal pressure was loaded at 1.2 MPa.

The representative internal pressure of 1.0 MPa was selected for analysis. The yield stress at pipe invert calculated by finite element simulation was 17263.30 kPa, which was 2409.88 kPa and 2118.31 kPa higher than the bonding assumption and smooth assumption.

The main reasons for the error in circumferential stress calculation are as follows: firstly, the thin-wall assumption was adopted in the calculation of the two theories, and the calculation of the bending moment of the cross-sectional was neglected. This part assumed that the influence of the bending moment distribution in the direction of the thickness direction on the pipe stress was ignored. The second reason was that the calculation of the constraint effect of soil on the ABR pipe in the above theory was biased. Converting the constraint effect of soil on the pipe into the load applied to the pipe will increase the stress on the critical section. Thirdly, the pipe-soil interaction was not considered when the traffic load was converted into soil pressure applied to the pipe. Hence, establishing the theory of pipe-soil interaction was necessary to explain the above errors, and the reasons for the errors will be the focus of the author’s subsequent research.

The above theories underestimated the maximum bearing capacity of the ABR pipe under the combined action of soil pressure, traffic load, and internal pressure, which was dangerous for engineering applications. In summary, the safety factor should be considered to evaluate the bearing capacity of the ABR pipe. The following formula expresses the corrected stress for buried ABR pipes:


$$\sigma_{ABR}^{\prime }=\text{1}.\text{14}\sigma_{ABR}$$ (16)

**Fig 21:**
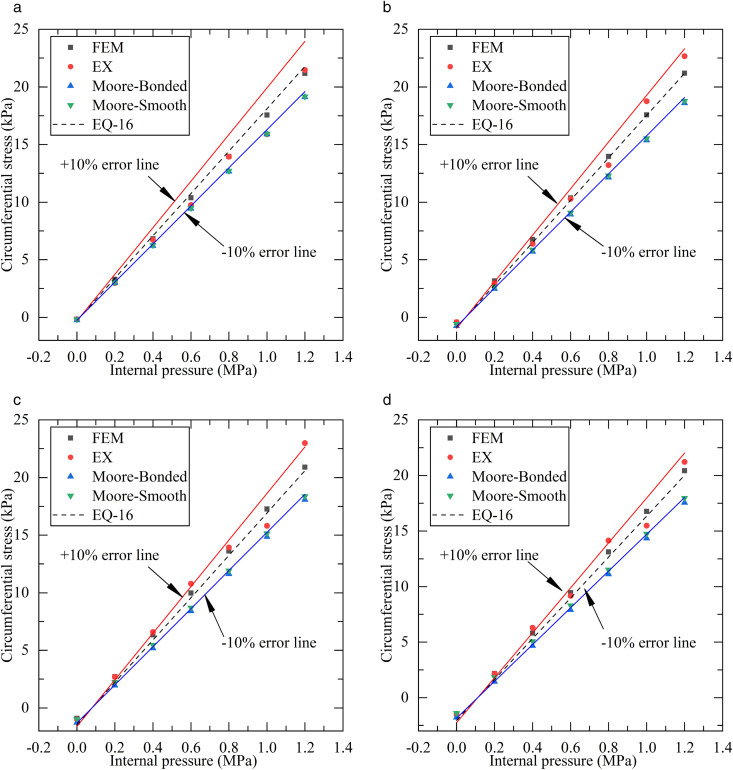
Results comparison and error analysis. (a) 0 **t. (b)** 5t. (c) 10 **t. (d)** 15t.

Taking the 90° position at the pipeline midspan and location-2 as an example, the circumferential stress of the pipeline cross-section was calculated using Equation (16). The results were compared with those obtained from finite element analysis, experimental measurements, and other theoretical methods, as shown in Fig 21. When a safety factor of 1.14 was applied, the calculated hoop stress demonstrated good agreement with FEA results, exhibiting minimal deviation. Similarly, small discrepancies were observed compared to most experimental measurements, though notable deviations occurred with individual test data. These deviations from experimental results primarily stemmed from measurement fluctuations inherent in the testing process.

## Discussion

The findings of this study align with several previous studies on buried pipelines under traffic loads and internal pressures. For instance, Rakitin and Xu [1] used centrifuge modeling to study large-diameter underground pipes subjected to heavy traffic loads and found that the stress distribution in the pipe was significantly influenced by the load position and magnitude. Similarly, Alzabeebee et al. [2] conducted a comparative study of buried pipes under static and moving loads and reported that the most critical sections of the pipe were typically located at specific angles, such as 90° and 180°, which is consistent with our results. Our experimental and numerical results further confirm that internal pressure has a more pronounced effect on the circumferential stress of the pipe than traffic load, which is supported by the findings of Robert et al. [3] and Li et al. [7].

However, there are also some differences between our study and previous research. For example, while many studies have focused on concrete and PE pipes, our work specifically investigates ABR pipes, which have unique material properties such as high toughness and impact resistance. Additionally, our study provides a more detailed analysis of the combined effects of traffic load and internal pressure, particularly in terms of the axial location and diameter-thickness ratio of the pipe.

Despite the comprehensive nature of this study, there are several limitations that should be addressed in future research:

1. Long-term Effects and Creep Behavior: The current study focuses on the short-term mechanical performance of buried ABR pipes. However, in real-world applications, buried pipelines are often subjected to long-term loads, which can lead to creep deformation and degradation of mechanical properties. Future research should investigate the long-term effects and creep behavior of ABR pipes under sustained traffic loads and internal pressures. This can be done through long-term monitoring experiments and accelerated aging tests.
2. Severe Overloading Conditions: The experimental conditions in this study were based on standard traffic load limits (e.g., 10 t axle load). However, in practice, buried pipelines may be subjected to severe overloading conditions, such as those caused by overweight vehicles or unexpected dynamic loads. Future research should explore the mechanical performance of ABR pipes under severe overloading conditions to better understand the potential risks and failure mechanisms.
3. Ground Subsidence and Soil-Pipe Interaction: Ground subsidence can significantly affect the stress distribution and mechanical performance of buried pipelines. The current study assumes a uniform soil foundation, but in reality, ground subsidence and uneven soil conditions can lead to complex soil-pipe interactions. Future research should incorporate ground subsidence scenarios and advanced soil-pipe interaction models to more accurately simulate real-world conditions.
4. Material Degradation and Environmental Factors: Polymer materials like ABR pipes can degrade over time due to environmental factors such as temperature variations, chemical exposure, and UV radiation. These degradation processes can reduce the mechanical performance of the pipes. Future studies should investigate the combined effects of mechanical loads and environmental factors on the long-term performance of ABR pipes.
5. Optimization of Pipe Design and Safety Factors: While this study proposes a theoretical model for calculating the circumferential stress of buried ABR pipes, further optimization of the model is needed to account for uncertainties and variabilities in real-world applications. Additionally, the safety factors used in engineering design should be refined based on more extensive experimental data and probabilistic analyses to ensure both safety and cost-effectiveness.

The experimental and numerical results obtained in this study provide valuable insights into the mechanical behavior of buried ABR pipes. The circumferential stress measurements at different angles and under various loading conditions reveal the critical sections and failure modes of the pipes. For example, the highest tensile stress was observed at 180° under combined traffic load and internal pressure, indicating that this location is the most vulnerable to failure. This finding is consistent with the numerical simulations, which also showed that the stress concentration at the bottom of the pipe is primarily influenced by internal pressure.

Comparing the experimental results with the finite element simulations, we found good agreement in terms of stress distribution and magnitude. The average error between the experimental and numerical results was approximately 5%, which is within an acceptable range for engineering applications. However, some discrepancies were observed, particularly at certain angles and under specific loading conditions. These differences may be attributed to factors such as simplifications in the numerical model (e.g., assuming uniform soil properties and neglecting micro-cracks in the pipe material) and variations in experimental conditions (e.g., soil compaction and moisture content). Future research should aim to reduce these discrepancies by refining the numerical models and improving the experimental setup.

## Conclusions

This study, using experimental and numerical approaches, offers comprehensive insights into the mechanical performance of buried ABR pipes under traffic load, internal pressure, and their combined effects. The main conclusions drawn from experiments, finite element simulations, and theoretical calculations are as follows:

1. The circumferential compressive and tensile stresses on buried ABR pipes were generated by traffic load and internal pressure, respectively, and internal pressure exerted a more dominant influence on circumferential stress than traffic loads. Under the traffic load alone, the most unfavorable angle for circumferential stress was 90°, while the unfavorable angle occurred at 180° under other loading conditions.
2. The most unfavorable axial location was directly beneath the center of the traffic load, with a horizontal influence distance of approximately 0.3 m under the diffusion pattern of the traffic load. The influence of traffic load can be neglected when the distance exceeds 0.3 m. With the wall thickness increased (i.e., reducing the diameter-to-thickness ratio), the cross-sectional area and circumferential stiffness of the ABR pipe were significantly enhanced. Consequently, circumferential tensile stress in the pipe was markedly decreased, with a maximum reduction of 68.17%.
3. The buried ABR pipeline exhibited superior performance under specific conditions of the 10 t traffic load, 1.0 MPa internal pressure, and 6.2 mm wall thickness. The circumferential stress at the bottom of the pipe was uniformly distributed along the axis and exceeded the stress at the top. The mechanical performance of the buried ABR pipe was primarily influenced by internal pressure.
4. The theoretical calculation model for circumferential stress in buried ABR pipes under soil, traffic, and internal pressure was established by combining the prism load method and Moore’s method. The significant advancement of the model was that the safety factor was proposed by comparing with the experiment values and finite element results, enhancing the safety and reliability of engineering design.

The results of this study have important practical implications for the design, construction, and maintenance of buried ABR pipe systems. By understanding the critical sections and stress distribution patterns of the pipes under different loading conditions, engineers can optimize the pipe design and select appropriate safety factors to enhance the reliability and service life of the pipelines. Additionally, the findings highlight the importance of proper backfilling and soil compaction practices to minimize the adverse effects of traffic loads and ensure the structural integrity of the pipes.

In summary, this study provides a solid foundation for the mechanical analysis and design of buried ABR pipes. However, further research is needed to address the limitations identified and to extend the findings to more complex and realistic scenarios. By building on the results of this study and incorporating advanced experimental and numerical techniques, future research can contribute to the development of more robust and durable buried pipeline systems.

## Supporting information

S1 File Data of experiment and numerical results. (ZIP)
